# A newborn with cardiomegaly

**DOI:** 10.4103/0974-2700.66541

**Published:** 2010

**Authors:** Shailendra Upadhyay, Sabrina Law, Dipak Kholwadwala

**Affiliations:** Schneider Children’s Hospital/Long Island Jewish Medical center, 269-01, 76^th^ Avenue, New Hyde Park, NY 115 01, USA

**Keywords:** Chest X-ray, Ebstein’s anomaly, electrocardiogram, inter-atrial communication, tricuspid valve

## Abstract

An infant with Down’s syndrome was noted to have hypoxemia and tachypnea at birth. The clinical examination, electrocardiogram (ECG) and the chest X-ray findings suggested a specific diagnosis that is not usually associated with Down’s syndrome. Despite the extremely rare association of Ebstein’s anomaly with Down’s syndrome, this diagnosis was highly suspected from the initial evaluation. An echocardiogram confirmed the diagnosis of Ebstein’s anomaly in this neonate. So far, only about seven cases of Ebstein’s anomaly associated with Down’s syndrome have been reported in the literature. This case is discussed for its rarity; it also highlights the importance of clinical examination and initial investigations that had suggested the diagnosis well prior to that of the echocardiogram.

## CASE REPORT

A 39-year-old mother delivered a full-term newborn female by emergency Cesarean section. The antenatal course was uncomplicated. Her Apgar scores were 6 at 1 minute and 9 at 5 minutes. The neonate was noted to have low trans-cutaneous oxygen saturations (88–92%) breathing room air, with mild tachypnea at a respiratory rate of 50–60 breaths per minute. The heart rate was in the range 110–140 beats per minute. Pertinent physical findings included features of Down’s syndrome, clear auscultatory lung findings, widely split first heart sound, a third heart sound and a grade 2/6 holosystolic murmur best appreciated along the right lower sternal border.

**Figure 1:**
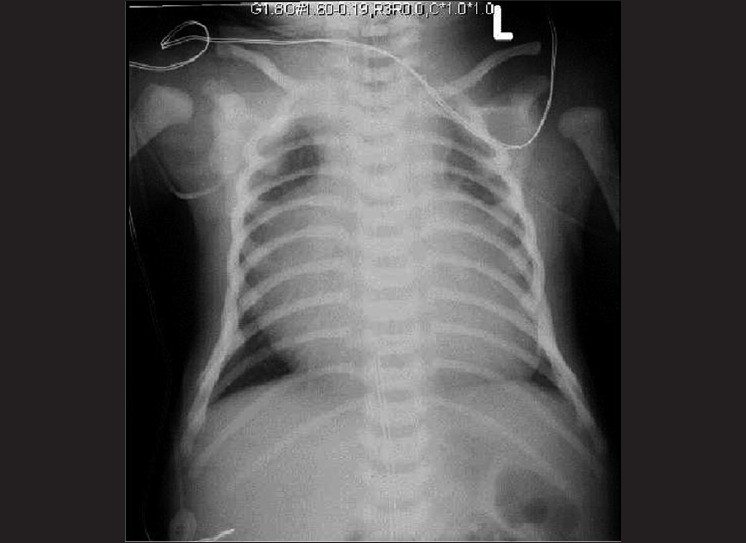
Chest X-ray demonstrating massive cardiomegaly with oligemic appearing lung fields

The chest X-ray [Figure 1] demonstrated massive cardiomegaly with oligemic appearing peripheral lung fields.

**Figure 2:**
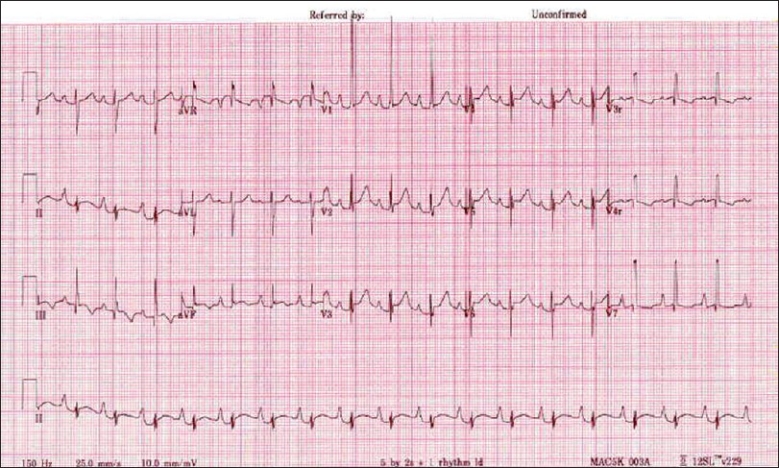
ECG demonstrating a normal sinus rhythm at 106 beats per minute; large “P” waves indicating right atrial enlargement with a first degree AV block for age (PR interval = 180 ms)

The electrocardiogram (ECG) [Figure 2] demonstrated a normal sinus rhythm with first-degree atrio-ventricular block (for age). A significant right atrial enlargement and right ventricular enlargement were also noted. The arterial and venous blood gases were normal except for mildly decreased PO_2_ (55%) on arterial blood gas.

Cardiac anomalies that are most commonly associated with Down’s syndrome are endocardial cushion defects. However, the findings on physical examination, ECG and chest radiograph suggested the diagnosis of Ebstein’s anomaly.

**Figure 3:**
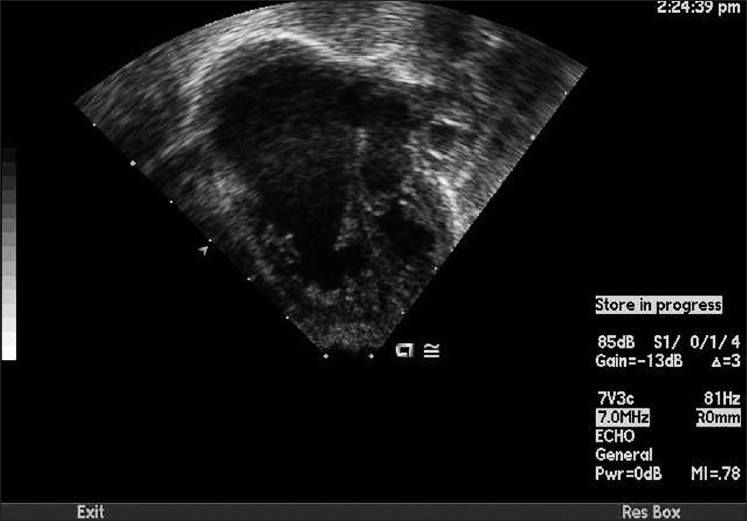
An apical four-chamber view demonstrating a massively dilated right atrium with distally displaced and dysplastic appearing tricuspid valve leaflets consistent with the Ebstein’s anomaly of the heart

An echocardiogram [Figure 3] confirmed the diagnosis of an Ebstein’s anomaly. It demonstrated a massively dilated right atrium with distal displacement of the tricuspid valve leaflets and a large inter-atrial communication with predominantly right to left shunting. Moderate to severe degree of tricuspid valve regurgitation was also noted. The tricuspid valve gradient was 42 mm Hg with the systemic blood pressure of 70/40 mm Hg. Assuming the right atrial pressure to be about 8 mm Hg, the right ventricular pressure was at 50 mm Hg (above half of systemic level).

The newborn was placed on nasal, continuous positive airway pressure (NCPAP) support and she clinically improved. Within the next few days, the NCPAP and oxygen support were gradually weaned off. The trans-cutaneous oxygen saturations normalized to over 95% range on room air. The follow-up echocardiogram demonstrated a left to right shunting across the inter-atrial communication and the estimated right ventricular pressures were below half of the systemic levels. Most newborns with mild to moderate degree of Ebstein’s anomaly would gradually recover over the next few weeks of life with a decrease in pulmonary vascular resistance (PVR). The decrease in the PVR will decrease the right to left shunting across the inter-atrial communication, thereby decreasing the cyanosis. Out patient who followed this typical course however needed further support in the newborn ICU for feeding issues. A cardiology follow-up was scheduled in a few weeks to re-evaluate, as the PVR would have decreased further.

## DISCUSSION

Down’s syndrome is most commonly associated with cardiac defects such as a ventricular septal defect, and an endocardial cushion defect. With features of Down’s syndrome in this neonate, these were the initially suspected cardiac defects.

The physical examination revealed a widely split first heart sound, from the delayed closure of tricuspid valve leaflets due to the Ebstein’s anomaly. A third heart sound was noted from rapid filling of the right ventricle. The murmur of tricuspid regurgitation was audible, along the right lower sternal border.

Chest X-ray demonstrated a massively enlarged heart with mostly clear appearing lung fields and the distal lung fields appeared oligemic. Conditions presenting with massive cardiomegaly on a chest X-ray in the neonatal period would include all cause fetal cardiomyopathy, vascular (celebral/hepatic) arterio-venous malformations (AVM) and Ebstein’s anomaly of the tricuspid valve. In the former two conditions, there would be bi-ventricular failure with pulmonary edema. The lung fields on chest X-ray, therefore, would show evidence of pulmonary vascular congestion. In an AVM, a continuous murmur may be audible over the cranium, lungs or the liver. No such murmur was appreciated in our patient. In patients with Ebstein’s anomaly, the left ventricular function is normal. The right atrium becomes massively dilated with atrialization of the right ventricle leading to a small right ventricle. With high PVR at birth and associated increase in right ventricular end diastolic pressure, shunting of blood occurs from right to left across the inter-atrial communication. This leads to cyanosis and desaturation. This is also responsible for oligemic appearance of the lung fields on chest X-ray. As the PVR falls, the right ventricular compliance improves and the shunt reverses left to right across the inter-atrial septum with improvement in cyanosis.[1]

The ECG demonstrated a first-degree AV block with significant right atrial enlargement. These findings were consistent with the Ebstein’s anomaly of the tricuspid valve.[2] Ebstein’s anomaly may be associated with accessory conduction pathways and Wolff-Parkinson-White (WPW) syndrome in up to 30% of patients. No evidence of ventricular pre-excitation or a supra-ventricular tachycardia was noted in our patient during her stay in the newborn ICU.

The echocardiogram confirmed the diagnosis of Ebstein’s anomaly. It revealed a massively dilated right atrium with distally displaced tricuspid valve leaflets. The inter-atrial communication revealed a right to left shunt at initial presentation.

Ebstein’s anomaly includes variable degree of malformation and displacement of the anterior leaflet with apical displacement of the septal and posterior tricuspid valve leaflets. This results in atrialization of the right ventricle. In 1866, Wilhelm Ebstein first described a patient with these features.[3]

Incidence of Ebstein’s anomaly of the tricuspid valve is approximately 1:20,000 live births, accounting for less than 1% of all congenital heart defects. Ebstein’s anomaly is therefore a rare disorder. Association of Ebstein’s anomaly with Down’s syndrome is extremely rare and only about six cases to date have been reported in the literature to the best of our knowledge.[4]

## CONCLUSION

This case illustrates the importance of clinical examination and basic investigations that may suggest the diagnosis of rare cardiac conditions such as an Ebstein’s anomaly. Such patients are likely to present at peripheral medical facilities managed by emergency room physicians. Systematic approach may lead the physicians to care for such patients at places where advanced investigation tools such as an echocardiogram may not be immediately available.
